# European Code Against Cancer, 5th edition – diet, excess body weight, physical activity, sedentary behavior, breastfeeding, and cancer

**DOI:** 10.1002/1878-0261.70201

**Published:** 2026-01-16

**Authors:** Michael F. Leitzmann, Ioanna Bakogianni, Annie S. Anderson, Linda Bauld, Esteve Fernandez, Sherry Morris, Bernard Srour, Constantine Vardavas, Ioana Vlad, Sabine Vuik, Matty Weijenberg, Marta Roqué I Figuls, David Rigau, Ariadna Feliu, Hajo Zeeb, Joachim Schüz, Erica D'Souza, David Ritchie, Carolina Espina, Elio Riboli

**Affiliations:** ^1^ Department of Epidemiology and Preventive Medicine University of Regensburg Germany; ^2^ Joint Research Centre – European Commission Ispra Italy; ^3^ Division of Population Health & Genomics, Ninewells Medical School University of Dundee Scotland; ^4^ Usher Institute and Behavioural Research UK University of Edinburgh Scotland; ^5^ Tobacco Control Unit WHO Collaborating Centre for Tobacco Control, Catalan Institute of Oncology (ICO) L'Hospitalet de Llobregat Spain; ^6^ Tobacco Control Research Group Institut d'Investigació Mèdica de Bellvitge – IDIBELL L'Hospitalet de Llobregat Spain; ^7^ School of Medicine and Health Sciences, Campus de Bellvitge University of Barcelona L'Hospitalet del Llobregat Spain; ^8^ Secretariat of Public Health, Department of Health Generalitat de Catalunya Barcelona Spain; ^9^ Imperial College London London UK; ^10^ Université Sorbonne Paris Nord and Université Paris Cité, INSERM, INRAE, CNAM, Center of Research in Epidemiology and StatisticS (CRESS), Nutritional Epidemiology Research Team (EREN) Bobigny France; ^11^ Nutrition and Cancer Research Network (NACRe Network) Jouy‐en‐Josas France; ^12^ Department of Hygiene, Epidemiology and Medical Statistics, School of Medicine National and Kapodistrian University of Athens Greece; ^13^ Department of Oral Health Policy and Epidemiology Harvard School of Dental Medicine Boston Massachusetts USA; ^14^ Department of Policy and Public Affairs World Cancer Research Fund International London UK; ^15^ Organisation for Economic Co‐operation and Development Paris France; ^16^ Department of Epidemiology, GROW Research Institute for Oncology and Reproduction Maastricht University The Netherlands; ^17^ Institut de Recerca Sant Pau (IR SANT PAU), CIBERESP, Cochrane Iberoamérica Barcelona Spain; ^18^ Department of Primary Care and Public Health, School of Public Health Imperial College London UK; ^19^ Environmental and Lifestyle Epidemiology Branch International Agency for Research on Cancer Lyon France; ^20^ Department of Prevention and Evaluation Leibniz ‐ Institute for Prevention Research and Epidemiology ‐ BIPS GmbH Bremen Germany; ^21^ Cancer Epidemiology and Prevention Research Unit Imperial College London UK

**Keywords:** body weight, breastfeeding, cancer, diet, European Code Against Cancer, physical activity, sedentary behavior

## Abstract

Diet, body weight, physical activity, sedentary behavior, and breastfeeding are modifiable factors influencing the cancer burden in the European Union, shaped by underlying social, commercial, environmental, and behavioral conditions. Excess body weight has reached epidemic levels, significantly influenced by widespread intake of sugar‐sweetened beverages and ultra‐processed foods rich in sugar, fat, and salt. Consumption of red and processed meat also commonly exceeds dietary recommendations. Physical inactivity and prolonged sedentary behavior are widespread. Breastfeeding rates vary widely across Europe but are generally low, particularly in high‐income countries. To reduce cancer risk, the European Code Against Cancer, 5th edition (ECAC5) recommends a diet rich in whole grains, vegetables, legumes, and fruits, while limiting red meat and avoiding processed meat. Intake of vegetables, legumes, and fruits prevents aerodigestive tract cancers, while diets high in whole grains and low in red and processed meat reduce colorectal cancer risk. Avoiding excess body weight through diet and physical activity, and limiting prolonged sitting, decreases risk of numerous cancers. Promoting and supporting sustained breastfeeding contributes to lowering breast cancer risk. Key policy interventions, such as fiscal incentives, urban planning, marketing restrictions, and public awareness campaigns, are central to creating supportive environments for cancer prevention.

## Graphical abstract

As part of the 14 recommendations on cancer prevention included in the 5th edition of the European Code Against Cancer (ECAC5), we here update the cancer prevention recommendations on: diet, excess body weight, physical activity, sedentary behavior, and breastfeeding. Individuals are encouraged to take action by following a largely plant‐based diet, maintaining a healthy weight, being physically active, reducing sedentary time, and breastfeeding if possible. Policymakers are urged to support environments that make healthy choices easier, affordable, accessible, equitable, and sustainable for all.

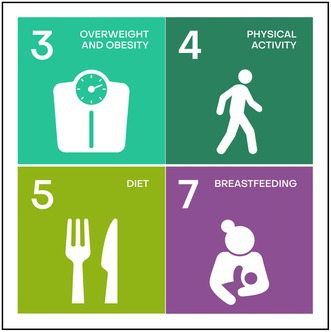

**Abbreviations**
AICR: American Institute for Cancer Research
BMI: Body mass index
EC: European Commission
ECAC: European Code Against Cancer
ECAC4: European Code Against Cancer, 4th edition
ECAC5: European Code Against Cancer, 5th edition
EFSA: European Food Safety Authority
EU: European Union
HaDEA: European Health and Digital Executive Agency
HCAs: Heterocyclic amines
HFSS: Foods High in Fat, Salt, and Sugar
IARC: International Agency for Research on Cancer
ICMJE: International Committee of Medical Journal Editors
IGF: Insulin‐like growth factor
NCD: Noncommunicable disease
PAF: Population attributable fraction
PAHs: Polycyclic aromatic hydrocarbons
SES: Socioeconomic status
SSB: Sugar‐sweetened beverage
UPF: Ultra‐processed food
WCRF: World Cancer Research Fund
WHO: World Health Organization

## 1 Introduction

**Fig. 1:**
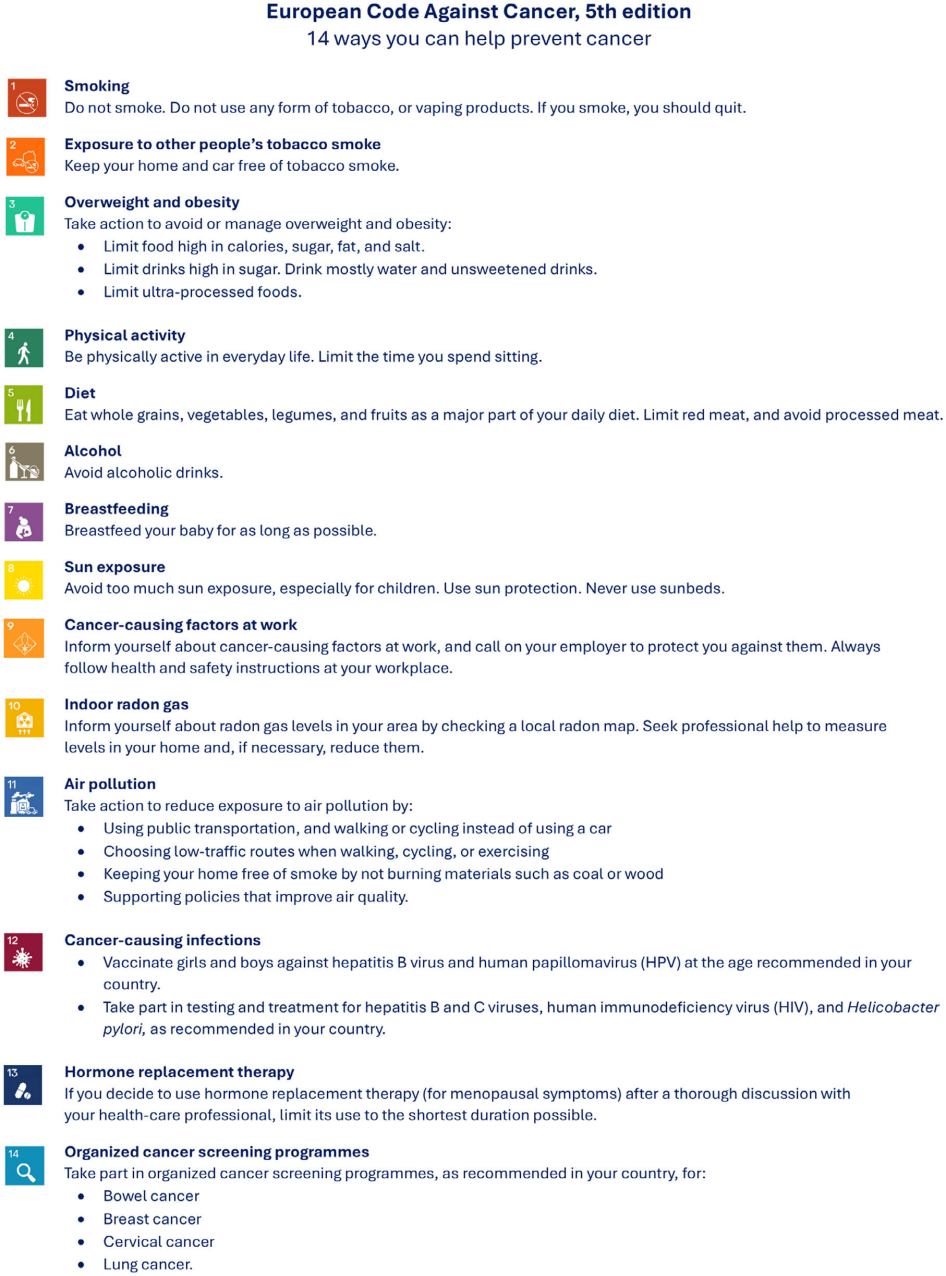
European Code Against Cancer, 5th edition: recommendations for individuals. The 14 recommendations of the European Code Against Cancer, 5th edition (ECAC5) adopted by the Scientific Committee of the ECAC5 project. © 2026 International Agency for Research on Cancer / WHO. Used with permission.

The European Code Against Cancer (ECAC) is a public health initiative established by the European Commission (EC) to provide evidence‐based recommendations for reducing cancer risk. The current 5th edition (ECAC5) [1] (Fig. 1) builds on ECAC4 [2] within the broader World Code Against Cancer Framework [3] introduced by the International Agency for Research on Cancer/World Health Organization (IARC/WHO) in 2022. This paper applies IARC's peer‐reviewed and consensus‐driven methodology [4] to review the latest evidence on diet, overweight and obesity, physical activity, sedentary behavior, and breastfeeding in relation to cancer and presents updated recommendations for the public, now complemented by population‐level measures aimed at policymakers, each supported by a summary of the underlying evidence (Annex S1).

## 2 Prevalence of risk and protective factors (diet, excess body weight, physical activity, sedentary behavior, and breastfeeding) within the European Union (EU)

### 2.1 Diet

Diets that deviate from established nutritional recommendations, characterized by low intake of fruits, vegetables, and dietary fiber and high intake of red and processed meat, sugar‐sweetened beverages, and ultra‐processed foods, remain widespread across Europe and contribute substantially to cancer risk. Globalization and rising living standards have led to increasingly similar diets, with Northern and Mediterranean patterns converging toward a Western‐style diet. This convergence involved both beneficial and detrimental shifts. For example, Northern countries now consume more fruits and vegetables than in the past, though intake remains suboptimal, while Mediterranean regions have increased their consumption of animal products. These trends began in Western Europe and later extended to Eastern Europe [5]. Although some changes point to modest dietary improvements, the broader shift toward Western‐style eating reflects a complex nutritional transition with mixed public health implications. Evidence from European populations suggests that women, older adults, and higher‐educated individuals are more likely to follow healthier diets, with greater consumption of fruits, vegetables, fish, and nuts, and less red and processed meat [6].

### 2.2 Excess body weight

Overweight and obesity have reached epidemic levels in the WHO European Region, affecting 59% of adults, with the highest rates in Mediterranean and Eastern European countries [7]. Obesity rose by 21% in the decade before 2016 and by 138% since 1975, while combined overweight and obesity increased by 8% and 51%, respectively [8]. Childhood obesity is also rising, with 4.4 million children under age five (7.9%) affected in 2020, often leading to adult obesity and long‐term health risks [8, 9].

Overweight is more common in males (63%) than females (54%), though obesity is slightly higher in females (24% vs. 22%), reaching up to one‐third of adults in some countries [7]. In high‐income countries, obesity is inversely linked to socioeconomic status (SES), while in low‐income settings, the relationship is positive, with mixed patterns in middle‐income countries [10]. Social and psychological factors, like stress, neighborhood conditions, self‐esteem, and early adversity, also play a role, especially in women [11]. Some evidence suggests obesity may, in turn, reduce SES through discrimination and increased health‐care costs [12]. The role of the built environment, including walkability and infrastructure, remains mixed [13], while broader factors such as air pollution, occupational exposures, and endocrine disruptors are increasingly implicated in metabolic dysfunction. An overarching driver is the obesogenic environment, shaped by structural forces including food marketing, labor policies, and economic inequality, which reinforce and exacerbate health disparities.

### 2.3 Physical activity

In the EU, physical inactivity remains widespread, with 35% of adults insufficiently active in 2016 [14]. However, participation in nonsport activities, such as cycling, dancing, and gardening, increased from 44% to 53% between 2017 and 2022. Nordic countries report the highest engagement, Western Europe intermediate levels, and Southern Europe the lowest, while Eastern Europe shows mixed participation. Walking generally follows overall activity trends, but some regions deviate: Parts of Western Europe show low walking rates alongside moderate recreational activity, while certain Southern and Eastern regions report frequent walking despite low recreational activity [15].

Physical activity, defined as ever engaging in nonsport activities (regularly, with some regularity, or seldom), is more common among men than women (72% vs. 65%) and declines with age, from 84% among 15 to 24‐year‐olds to 60% among those 55 and older. Education and financial stability play key roles, with participation at 80% among higher‐educated individuals but only 45% among those with lower education. Financially stable individuals (74%) are more active than those facing frequent financial difficulties (52%) [15]. Higher SES individuals tend to be more active in recreational activities, but this pattern does not extend to other domains of activity, as transport, occupational, and household activity show weak or inverse associations with SES [16].

### 2.4 Sedentary behavior

Sedentary behavior is prevalent in the EU. Prolonged sitting (8.5 or more hours/day) is most common in Northern Europe (14%–26%), reflecting higher rates of sedentary work environments in this region, while Western Europe shows moderate levels (10%–15%). Eastern Europe has a lower prevalence (7%–13%), and Southern Europe reports the least (7%–9%), likely due to cultural factors favoring mobility [15]. From 2005 to 2017, sedentary behavior increased across most of Europe, with steady rises in Northern and Western regions, while Eastern and Southern Europe showed mixed trends. Some areas experienced sharp increases (9%–19%), while others experienced reductions [17].

Prolonged sitting is more common among younger individuals, declining from 13% at ages 15–24 to 10% at 55+. Prolonged sitting is also slightly more common among men than women (12% vs. 10%). Higher SES also correlates with extended sedentary time: 14% of highly educated vs. 8% of less educated individuals, and 16% of white‐collar vs. 5% of manual workers. Perceived social class shows a similar pattern, with 18% of those identifying as upper‐class sitting extensively, compared to 11% in lower‐income groups [15].

### 2.5 Breastfeeding

The WHO recommends exclusive breastfeeding for the first 6 months, introduction of complementary food thereafter, and continued breastfeeding up until 2 years or beyond [18]. Most European countries fall short of this, with inconsistent rates of early initiation, exclusive breastfeeding, and continued breastfeeding, and exclusive breastfeeding notably declining after 4 months [19]. Northern European countries generally report low exclusive breastfeeding rates at or under 6 months, with some as low as 1%, while others achieve slightly higher but still modest rates. In Western Europe, rates vary but remain mostly low to moderate, with some countries below 10% and others exceeding 20%. Southern Europe shows substantial variation, including some of the lowest rates in Europe, alongside a few higher‐performing countries reaching around 35%. Eastern Europe also varies substantially, with some nations at 2%–4%, others exceeding 40%, and a few surpassing 50%, among the highest in Europe [19].

## 3 Cancer burden in the EU/Europe attributable to unhealthy diet, excess body weight, physical inactivity and sedentary behavior, and breastfeeding

The burden attributable to unhealthy diet, including low fiber, fruit, and vegetable intake, and high consumption of processed and red meat, ranges from 4.5% to 10% for all cancers, with estimates of 10% in the Netherlands, 7.7% in Germany, 4.5%–6.3% in Italy, and 5%–5.4% in France [20, 21, 22, 23]. For excess body weight, population attributable fraction (PAF) estimates range from 3.6% to 7% across Europe: 7% in Germany, 5%–5.3% in France, 4%–5% in Finland, 4% in the Netherlands, and 3.6%–4% in Italy [20, 21, 23, 24, 25, 26]. Physical inactivity contributes to 0.8%–6% of cancers in Europe, with PAFs of 6% in Germany, 2% in the Netherlands, 2% in the United Kingdom, 1% in Nordic countries (for colon, endometrial, and postmenopausal breast cancer), and 0.8% in France [20, 21, 27, 28]. In the UK, 4.7% of breast cancer cases are attributable to not breastfeeding [29], while in France, breastfeeding for less than 6 months is linked to 3.2% of breast cancer and 8.6% of ovarian cancer cases [30].

## 4 Recommendations for individuals

The ECAC5 Lifestyle Determinants Working Group reviewed the latest evidence on diet, overweight and obesity, physical activity, sedentary behavior, breastfeeding, and cancer, updating ECAC4 recommendations using IARC methodology [4]. Each topic was evaluated through an Evidence‐to‐Decision process that explicitly considered equity, suitability, actionability, and acceptability within the European context by assessing whether recommendations were clear, culturally relevant, and feasible to implement under existing European health and policy frameworks, and whether they might reduce or widen health inequalities. Recommendations were retained or revised only if supported by sufficient evidence that a cause of cancer can be avoided or reduced, and/or an intervention proven effective [4]. This corresponds to the IARC Monographs and Handbooks grading of ‘sufficient’ and the World Cancer Research Fund/American Institute for Cancer Research (WCRF/AICR) grading of ‘convincing’ or ‘probable’ evidence [4, 31], thereby meeting the evidence threshold defined by both agencies. ECAC5 builds on this foundation by incorporating new evidence published since ECAC4, commissioning additional systematic reviews where needed, and adapting the recommendations to enhance their scientific rigor and practical applicability across the EU.

## 5 Scientific justification for update of the diet recommendation in ECAC5

The ECAC4 recommendation on maintaining a healthy diet to reduce personal cancer risk stated:

‘Have a healthy diet to reduce the risk of cancer: eat plenty of whole grains, pulses, vegetables, and fruits; limit high‐calorie foods (foods high in sugar or fat); avoid sugary drinks and processed meat; and limit red meat and foods high in salt.’ [32]

The ECAC5 diet recommendation remains largely unchanged in its core message:

*Eat whole grains, vegetables, legumes, and fruits as a major part of your daily diet*.

*Limit red meat and avoid processed meat*.

The main change in ECAC5 is the shift of recommendations on sugar, salt, fat, and high‐calorie foods from diet to the body weight section, highlighting their role in cancers related to excess body weight.

Whole grains, vegetables, legumes, and fruit are rich in fiber, which colonic microbiota convert into short‐chain fatty acids that may lower colorectal cancer risk by reducing carcinogen exposure and improving insulin sensitivity [33]. Vegetables also provide bioactive compounds (e.g., carotenoids, vitamins C/E, selenium, flavonoids) with anticarcinogenic effects. Fruits and nonstarchy vegetables contain polyphenols and carotenoids (e.g., beta‐carotene, lycopene) that support immunity and inhibit tumor growth [34]. High‐temperature cooking of meat produces carcinogens (heterocyclic amines, polycyclic aromatic hydrocarbons), while processed meats add nitro compounds and fats that raise secondary bile acid production. Heme iron in red meat promotes carcinogenic N‐nitroso compounds and oxidative stress, contributing to colorectal cancer risk [35].

## 6 Evidence on the association between diet and cancer, and benefits of following the recommendation

A diet high in whole grains and fiber is strongly linked to reduced colorectal cancer risk [36]. In contrast, red and processed meat increase risk [37, 38], with processed meat classified as a Group 1 carcinogen and red meat as Group 2A [39]. The WCRF/AICR rates the evidence as ‘convincing’ for processed meat and ‘probable’ for red meat; accordingly, prioritizing whole grains, vegetables, legumes, and fruits, while limiting red meat and avoiding processed meat, can help reduce cancer risk [40]. These plant‐based foods are also associated with lower risk of excess body weight, suggesting an additional, indirect pathway for cancer prevention [41].

## 7 Presentation of the diet recommendation

### 7.1 Equity

Promoting plant‐based diets while reducing red and processed meat can lower cancer risk, improve public health, and reduce health disparities. Diets rich in fruits, vegetables, and whole grains are linked to lower cancer risk across all SES groups [42]. However, access to affordable, diverse plant‐based foods remains limited for low‐income populations [43], requiring policies to improve affordability and availability.

### 7.2 Suitability, actionability, and acceptability of the recommendations for the individual

Plant‐based diets are highly relevant in Europe, where meat intake often exceeds recommendations [44]. The dietary guidance is actionable and aligns with EU dietary goals. Acceptability varies by age, gender, and cultural norms. While younger, sustainability‐driven individuals are more receptive, others, particularly those with lower SES, may face barriers such as limited time for meal preparation and lack of knowledge on how to prepare vegetables and salads. Education, labelling, and practical cooking guidance can improve acceptance [45].

## 8 Factors excluded from current diet recommendation

Certain dietary factors were not included in the final recommendation because the evidence was insufficient, inconsistent, or not relevant to the European context. Dairy products were not included because the evidence is heterogeneous, suggesting potential protection against colorectal cancer but a possible increased risk of prostate cancer, and thus does not support a uniform recommendation. Aflatoxin, a liver cancer risk factor, is omitted due to low EU exposure under strict food safety standards, though imported nuts and cereals remain a concern [46, 47]. Salted foods and high sodium intake, linked to gastric damage and stomach cancer, especially in the presence of *Helicobacter pylori*, are also not assessed [48, 49, 50]. Dietary supplements are excluded due to heterogeneous evidence, with high‐dose beta‐carotene increasing lung cancer risk in smokers [51], and calcium possibly reducing colorectal cancer risk [52]. Alcohol is not discussed here because it is comprehensively addressed in a separate ECAC5 publication developed by a dedicated working group [53].

## 9 Scientific justification for update of the excess body weight recommendation in ECAC5

The recommendation in ECAC4 was: ‘Take action to be a healthy body weight’ [54]. In ECAC5, it was modified to:

Take action to avoid or manage overweight and obesity:

- Limit food high in calories, sugar, fat, and salt.
- Limit drinks high in sugar. Drink mostly water and unsweetened drinks.
- Limit ultra‐processed foods.

The revised wording shifts focus from the subjective term ‘healthy weight’ to addressing excess body weight as a modifiable cancer risk factor. Although obesity has complex biological, social, and environmental determinants, it is regarded as modifiable in the ECAC5 framework because its main drivers, diet, physical activity, and sedentary behavior, can be shaped through behavioral and structural measures. ‘Avoid or manage overweight and obesity’ offers inclusive guidance while avoiding weight stigma. Limits on food high in calories, sugar, salt, fat, and ultra‐processed foods (UPFs) highlight their role in cancer risk beyond weight management, with strong evidence from WCRF/AICR linking sugar‐sweetened beverages, ‘fast foods’ and ‘Western type’ diets to increased risk of weight gain and excess adiposity [41], which are themselves indirect pathways to cancer. UPFs are industrial formulations with ingredients not used in home cooking, designed for convenience, palatability, and shelf life [55], that are mostly energy‐dense and nutrient‐poor, and have been linked to weight gain and excess adiposity [56, 57, 58]. Evidence for direct carcinogenic effects of food processing is limited, with the exception of processed meat, which has been classified by IARC as a cause of colorectal cancer [39].

Excess body fat increases cancer risk, with weight gain that exceeds healthy developmental or restorative changes compounding risk over the life course. High‐calorie intake, mainly from sugar and fat, drives weight gain [59]. Excess adiposity promotes a pro‐carcinogenic environment through insulin resistance, elevated estrogen, and chronic inflammation [60].

## 10 Evidence on the association between excess weight and cancer, and benefits of following the recommendation

Excess weight is a significant, modifiable cancer risk factor. Overweight and excess body fatness in adulthood increase the risk of cancers of the mouth, pharynx, larynx, esophagus (adenocarcinoma), stomach cardia, pancreas, gallbladder, liver, colon, rectum, postmenopausal breast, ovaries, endometrium, advanced prostate, and kidney [61]. Higher adiposity in childhood, adolescence, or young adulthood is causally linked to a modest increase in colorectal cancer risk [62]. In contrast, early life adiposity is related to a modest reduction in breast cancer risk at all ages [63]. Nonetheless, preventing excess adiposity remains a key recommendation due to its broad adverse health effects.

Evidence suggests intentional weight loss may reduce cancer risk in overweight individuals [64]. A systematic review commissioned by the ECAC5 Lifestyle Determinants Working Group found that sustained weight loss through lifestyle changes may slightly lower cancers related to excess body weight (moderate‐certainty evidence), though effects on specific cancers and overall incidence remain uncertain (low to very low‐certainty evidence) [65].

Evidence‐based strategies, such as limiting high‐calorie and sugary foods, choosing fresh, minimally processed options, eating dietary fiber, fruit, vegetables and whole grains, drinking mainly water, and staying physically active, can help reduce cancer risk related to excess body weight and support healthier lifestyles across the EU.

## 11 Presentation of the excess weight recommendation

### 11.1 Equity

In Europe, excess weight disproportionately affects lower SES populations, especially women [66]. To prevent widening health inequalities, recommendations should support individuals through policy and practice, rather than place the burden solely on them. Ensuring equitable access to affordable, nutrient‐dense foods, like fruits, vegetables, and whole grains, is essential, as these are often less available in low‐income areas. Policies such as healthy food subsidies and improved infrastructure can enhance access [67], while expanding parks and green spaces promotes physical activity [68]. Addressing structural barriers makes guidance more inclusive and effective. Further implementation research is needed to identify effective and equitable strategies for translating dietary and lifestyle recommendations into policy and practice across diverse European population groups.

### 11.2 Suitability, actionability, and acceptability of the recommendations for the individual

The recommendations are well‐suited across the EU, supporting a balanced diet and physical activity while respecting cultural preferences. They address both underweight and overweight concerns, promoting healthy weight without encouraging disordered eating. Acceptability relies on delivering advice in a supportive, nonstigmatizing way.

## 12 Scientific justification for update of the recommendation on physical activity and sedentary behavior in ECAC5

The recommendation on physical activity and sedentary behavior for reducing personal cancer risk in ECAC4 [69] remained unchanged in ECAC5:

*Be physically active in everyday life. Limit the time you spend sitting*.

Physical activity is associated with reduced cancer risk through various biological mechanisms, including improved sex hormone profiles, reduced chronic inflammation, decreased insulin resistance, enhanced immune function, oxidative stress modulation, and increased DNA repair capacity [70]. While high‐intensity exercise may transiently increase reactive oxygen species (ROS) and oxidative DNA damage [71], it also stimulates compensatory DNA repair processes, resulting in a net protective effect [72].

Prolonged sedentary behavior is increasingly recognized as a significant cancer risk factor, both directly and indirectly [73]. Indirect mechanisms include excess adiposity‐related factors, such as insulin resistance, disruptions in the insulin‐like growth factor (IGF) axis, and chronic systemic inflammation [74]. Emerging evidence highlights a direct relationship between sedentary behavior and cancer, as well as a newly identified link with cancer mortality [73], an endpoint not considered in ECAC4.

## 13 Evidence on the relations of physical activity and sedentary behavior to cancer, and benefits of following the recommendation

Higher physical activity is linked to lower risks of colon, postmenopausal breast, and endometrial cancers, with vigorous activity also reducing pre‐ and postmenopausal breast cancer risk. Emerging evidence suggests protective effects for esophageal, lung, liver, and premenopausal breast cancers [31]. Regular walking, a widely accessible activity, helps prevent excess adiposity, a known cancer risk factor [75]. Screen time is associated with excess body weight in children [76], with weaker evidence in adults [77]. Prolonged sedentary behavior is now recognized as an independent cancer risk factor [73]. Promoting physical activity and reducing sedentary time are key strategies for cancer prevention.

## 14 Presentation of the physical activity and sedentary behavior recommendation

### 14.1 Equity

In the EU, leisure‐time physical activity is more common among higher SES groups, while lower SES groups engage more in occupational activity [78]. Promoting affordable, accessible leisure‐time activity in underserved communities is key to reducing cancer disparities. Policies enabling active breaks, flexible work, and tailored information and education on sedentary risks can improve equity [79]. Physical activity recommendations should consider barriers like cost, time, and safe space access to avoid widening health inequalities.

### 14.2 Suitability, actionability, and acceptability of the recommendation for the individual

Physical activity guidelines are suitable for all age groups and abilities [80]. They are actionable, promoting everyday activities like walking or gardening and reducing sedentary time through standing breaks and screen time limits. Acceptability varies by culture, responsibilities, and norms. Culturally tailored strategies and workplace interventions can enhance adherence.

## 15 Scientific justification for update of the recommendation on breastfeeding in ECAC5

In ECAC4, the recommendation on breastfeeding to reduce personal cancer risk was as follows: ‘Breastfeeding reduces the mother's cancer risk. If you can, breastfeed your baby.’ [81]. In ECAC5, this has been updated to:

*Breastfeed your baby for as long as possible*.

The revised recommendation highlights that breastfeeding's protective effect increases with duration: ‘the longer, the better’ [31], reflecting strong evidence with no upper benefit limit. The phrase ‘if you can’ was removed for consistency, as it could apply to any behavior change advice.

Extensive evidence shows breastfeeding reduces breast cancer risk in mothers, primarily by lowering lifetime estrogen exposure and promoting healthy breast tissue renewal. There is also suggestive evidence of protection against ovarian cancer, possibly through prolonged lactational amenorrhea and reduced lifetime exposure to estradiol [82]. For infants, breastfeeding supports healthy development and offers protection against infections, allergies, asthma, and type 1 diabetes [83].

## 16 Evidence on the association between breastfeeding and cancer, and benefits of following the recommendation

Breastfeeding reduces overall breast cancer risk, with greater protection linked to longer duration [84]. The effect varies by receptor status; ever breastfeeding is associated with lower risk of ER‐/PR‐negative and especially triple‐negative breast cancers [85], which are more aggressive and common in younger women. No consistent link exists for hormone receptor‐positive cancers. There is also suggestive evidence that breastfeeding reduces ovarian cancer risk [82]. Having been breastfed also protects against weight gain, overweight, and excess body weight later in life, thereby providing an indirect benefit against cancer [31].

## 17 Presentation of the breastfeeding recommendation

### 17.1 Equity

Breastfeeding is shaped by a combination of social, cultural, economic, and biological factors and is more prevalent among higher SES women [86, 87, 88]. Targeted support is essential to reduce disparities and avoid stigmatizing vulnerable groups. Although higher SES women have higher breast cancer incidence due to factors like reproductive history, hormone replacement therapy, higher alcohol consumption, and greater access to screening, lower SES women face worse prognoses. Breastfeeding's protective effect is consistent across age, ethnicity, and reproductive history, and promoting it among lower SES groups can help reduce inequalities and support prevention.

### 17.2 Suitability, actionability, and acceptability of the recommendation for the individual

Breastfeeding is biologically suitable for most, though challenges like milk supply, mental health, cultural norms, and the availability of support can hinder practice. Actionability relies on support measures such as lactation counseling and workplace accommodations. Acceptability varies across EU populations, shaped by cultural and social factors. Policies that normalize and support breastfeeding are key to improving uptake and reducing stigma [89].

## 18 Co‐benefits for prevention of noncommunicable diseases (NCDs) other than cancer with similar risk factors and opportunities for health promotion

Many NCDs share risk factors with cancer, including unhealthy diet, excess body weight, physical inactivity, sedentary behavior, and lack of breastfeeding. Addressing these factors not only reduces cancer risk but also improves overall public health.

Diets high in whole grains, vegetables, legumes, and fruits reduce the risk of type 2 diabetes, metabolic syndrome, and cardiovascular disease [90, 91]. The Mediterranean diet, emphasizing these foods and limiting red meat and avoiding processed meat, is linked to lower risks of diabetes, heart disease, stroke, and cardiovascular mortality [92, 93]. Red meat consumption is also associated with heart failure and gestational diabetes [94]. Following WCRF/AICR dietary guidance lowers overall mortality risk [95].

Excess body weight increases the risk of diabetes, cardiovascular disease, hypertension, osteoarthritis, sleep apnea, fatty liver disease, and systemic inflammation [96]. Modest fat loss and maintaining a lower‐normal BMI significantly reduce these risks [97].

Regular physical activity reduces the risk of heart disease, stroke, diabetes, and mental health conditions, while improving immunity, fitness, and sleep [98]. Less sedentary time further supports prevention of chronic conditions, including diabetes, cardiovascular disease, and cancer [99].

Breastfeeding protects against early life infections [82], supports healthy growth, reduces risk of excess body weight in childhood, and improves maternal long‐term weight and NCD outcomes. It also lowers maternal risk of type 2 diabetes and cardiovascular disease [31].

## 19 Recommendations for policymakers

The ECAC5 policy recommendations build on, but go beyond, existing EU and WHO frameworks by identifying priority areas where further action, harmonization, and stronger implementation is needed [4]. While many EU policies already promote healthier diets, physical activity, and breastfeeding, their coverage, enforcement, and equity impact remain uneven across Member States. ECAC5 therefore emphasizes the need for integrated, cross‐sectoral strategies that make healthy choices easier and more affordable for all, strengthen the regulation of unhealthy products, and address social and environmental determinants of cancer risk.

## 20 Diet

Governments can promote healthier diets by ensuring access to safe drinking water and making nutritious foods, such as whole grains, vegetables, legumes, and fruit, the most affordable and accessible options in all settings. This also includes removing sugar‐sweetened beverages (SSBs) and high‐fat, salt, and sugar (HFSS) snacks from vending machines. Fiscal policies should raise taxes on unhealthy foods and reduce them on healthier options, creating strong economic incentives. Current evidence and expert consensus support public procurement standards limiting sugar, sodium, and red or processed meat; a harmonized EU front‐of‐pack label to aid low‐literacy populations; and stricter marketing controls on HFSS foods, especially for children. National dietary guidelines must be regularly updated and promoted, and nutrition education integrated into school curricula, including guidance on when the use of dietary supplements is justified, along with the associated risks, to prevent misuse and ensure evidence‐based practice. Public health campaigns should raise awareness about the excess body weight‐cancer link (Table 1) [100, 101, 102].

**Table 1:** European Code Against Cancer, 5th edition: recommendations for policymakers on diet.

Diet	
**Implement fiscal policies:**	
o	Increase taxes and prices of processed meat
o	Lower taxes and prices of whole‐grain products, vegetables, and fruit
**Make the healthy choice the easiest—most affordable, accessible, and available—option in all settings:**	
o	Implement procurement policies with mandatory standards that limit red and processed meat in all settings
o	Increase the availability, visibility, and affordability of whole‐grain products, vegetables, legumes, and fruit
o	Set mandatory standards that limit or ban foods high in sugars, fat, or salt
**Agree upon and implement an effective EU‐wide front‐of‐pack nutrition labelling scheme that is understood by all consumers**	
**Update and promote national food‐based dietary guidelines**	
**Update curricula to include nutrition education classes across the EU**	
**Complementing the above‐mentioned policy measures, implement regular public health campaigns to raise awareness of the importance of healthy nutrition in the prevention of cancer**	

© 2026 International Agency for Research on Cancer / WHO. Used with permission.

References: • European Union Action Plan on Childhood Obesity 2014–2020. Brussels: European Commission; 2014. Available from: https://health.ec.europa.eu/publications/eu-action-plan-childhood-obesity-2014-2020 [100]. • Commission Delegated Regulation (EU) 2017/40 of 3 November 2016 supplementing Regulation (EU) No 1308/2013 of the European Parliament and of the Council with regard to the Union aid scheme for the supply of fruit and vegetables, bananas and milk in educational establishments and amending Commission Implementing Regulation (EU) No 907/2014.*OJEU*. 2017;**L5**:11–22. Available from: https://eur-lex.europa.eu/eli/reg_del/2017/40/oj [101]. • Food‐Based Dietary Guidelines in Europe. Brussels: European Commission. Available from: https://knowledge4policy.ec.europa.eu/health-promotion-knowledge-gateway/topic/food-based-dietary-guidelines-europe [102].

### 20.1 Key policies

Several EU policies underpin efforts to promote healthier eating. Directive 2010/13/EU limits children's exposure to HFSS food marketing [103]. The EU Action Plan on Childhood Obesity encourages healthy eating and active lifestyles [100], reinforced by the EU School Fruit, Vegetables, and Milk Scheme, which introduces children to nutritious foods early in life [104]. The European Child Guarantee promotes health equity by ensuring vulnerable children have access to adequate nutrition [105]. Culturally tailored Food‐Based Dietary Guidelines underpin national nutrition policies [102], while front‐of‐pack labels help consumers make healthier choices [106]. The recommendations for policymakers are also supported by Europe's Beating Cancer Plan [107] and the WHO NCD best buys [108].

### 20.2 Creating enabling environments

Making healthy options more accessible than unhealthy ones requires a multi‐pronged approach. Strengthening public procurement in schools, workplaces, and hospitals ensures access to nutritious meals and limits HFSS foods. Fiscal measures can lower the cost of healthy foods and discourage ultra‐processed and sugary products. Clear, user‐friendly front‐of‐pack labels support informed choices, especially among those with limited health literacy [106]. Schools can foster healthy behaviors through nutrition education and practical activities like cooking and gardening. Robust dietary supplement regulations prevent deceptive marketing and ensure accurate health information [109].

### 20.3 Feasibility and resources

Several EU initiatives illustrate the success of policy‐driven approaches to promote and support healthier eating. Hungary's Public Health Product Tax has reduced HFSS consumption while generating revenue for public health initiatives [110]. France's Nutri‐Score labelling system has improved consumer awareness of food quality [111]. Finland's Salt Reduction Program lowered national sodium intake through product reformulation and public education [112]. In Italy, integrating nutrition education into school curricula has the potential to encourage healthier dietary habits from an early age [113]. Collectively, these initiatives underscore the value of coordinated dietary public health policies.

## 21 Excess body weight

Promoting healthy body weight builds on dietary policies that improve access to nutritious foods and reduce exposure to unhealthy options (see diet section), including fiscal measures, vending machine reforms, procurement standards, front‐of‐pack labelling, and marketing restrictions. To complement these structural measures, accessible and affordable weight management interventions are essential, particularly for underserved groups. These should be integrated across primary care, workplaces, and communities. Schools also play a key role by including nutrition education in curricula. Regular public health campaigns should raise awareness about the health risks of excess body weight, including its link to cancer, and highlight the importance of prevention across life stages (Table 2) [100, 101, 102, 103, 114].

**Table 2:** European Code Against Cancer, 5th edition: recommendations for policymakers on excess body weight.

Overweight and obesity	
**Implement fiscal policies:**	
o	Increase taxes and prices of foods high in sugars, fat, or salt, including processed meat, and sugar‐sweetened beverages
o	Lower taxes and prices of whole‐grain products, vegetables, legumes, and fruit
**Make the healthy choice the easiest—most affordable, accessible, and available—option in all settings:**	
o	Increase the availability, visibility, and affordability of whole‐grain products, vegetables, legumes, and fruit
o	Remove snacks high in sugars, fat, or salt and sweetened beverages from vending machines and other locations. Make the sugar‐free option the default option for hot beverages in vending machines
o	Provide fresh filtered tap water in all settings
**Implement procurement policies with mandatory standards that limit foods high in sugars, fat, or salt, including processed meat and sugar‐sweetened beverages in all settings. Specify upper limits for total daily energy from sugars and an upper limit of sodium intake per meal**	
**Ban or restrict marketing, advertising, and promotion of foods high in sugars, fat, or salt, especially to children**	
**Introduce and promote weight management interventions that are accessible and affordable for all citizens**	
**Agree upon and implement an effective EU‐wide front‐of‐pack nutrition labelling scheme that is understood by all consumers**	
**Update and promote national food‐based dietary guidelines**	
**Update curricula to include nutrition education classes across the EU**	
**Complementing the above‐mentioned policy measures, implement regular public health campaigns to raise awareness of the association of excess body weight with cancer risk and the importance of prevention of overweight and obesity**	

© 2026 International Agency for Research on Cancer / WHO. Used with permission.

References: • Directive 2010/13/EU of 10 March 2010 on the coordination of certain provisions laid down by law, regulation or administrative action in Member States concerning the provision of audiovisual media services (Audiovisual Media Services Directive). *OJEU*. 2010;**L95**:1–24. Available from: https://eur-lex.europa.eu/eli/dir/2010/13/oj [103]. • European Union Action Plan on Childhood Obesity 2014–2020. Brussels: European Commission; 2014. Available from: https://health.ec.europa.eu/publications/eu-action-plan-childhood-obesity-2014-2020 [100]. • Commission Delegated Regulation (EU) 2017/40 of 3 November 2016 supplementing Regulation (EU) No 1308/2013 of the European Parliament and of the Council with regard to the Union aid scheme for the supply of fruit and vegetables, bananas and milk in educational establishments and amending Commission Implementing Regulation (EU) No 907/2014. *OJEU*. 2017;**L5**:11–22. Available from: https://eur-lex.europa.eu/eli/reg_del/2017/40/oj [101]. • Food‐Based Dietary Guidelines in Europe. Brussels: European Commission. Available from: https://knowledge4policy.ec.europa.eu/health-promotion-knowledge-gateway/topic/food-based-dietary-guidelines-europe [102]. • Global report on the use of sugar‐sweetened beverage taxes. Geneva: World Health Organization; 2023. Available from: https://www.who.int/publications/i/item/9789240084995 [114].

### 21.1 Key policies

Efforts to address excess body weight build on the core policy frameworks outlined in the diet section. Existing EU initiatives, such as restrictions on HFSS food marketing to children [103], the EU Action Plan on Childhood Obesity [100], the School Fruit, Vegetables, and Milk Scheme [104], and the European Child Guarantee [105], promote healthier food environments and equitable nutrition access. The 2010 Council Conclusions on Reducing Salt Intake encourage national policies to lower population sodium consumption, a key contributor to diseases related to excess body weight [115]. The WHO Global Report on Sugar‐Sweetened Beverage Taxes also highlights the effectiveness of fiscal measures in reducing excessive sugar intake and the prevalence of excess body weight [114].

### 21.2 Creating enabling environments

Transforming environments to support healthy weight involves reinforcing dietary policy measures (see diet section) alongside targeted interventions. Schools, workplaces, and health‐care settings should offer nutritious foods and limit HFSS products by applying procurement standards that cap processed meat, SSBs, and sugar and sodium per meal. Additional measures, such as removing unhealthy vending items, making sugar‐free beverages the default, and improving access to drinking water, further support healthier daily choices. Fiscal strategies, including subsidies for fresh produce and taxes on unhealthy foods, remain key. Regularly updated dietary guidelines reinforce informed food choices [102]. Beyond environmental measures, affordable and accessible weight management interventions are essential, particularly for underserved populations, to support long‐term weight maintenance.

### 21.3 Feasibility and resources

Several EU countries have successfully implemented policies to address healthy weight. Examples include Hungary's Public Health Product Tax [110], France's Nutri‐Score labelling system [111], and Finland's salt reduction strategy [112]. These efforts, described in the diet section, illustrate how integrated fiscal and regulatory interventions have been implemented across EU Member States to promote healthier food environments, with preliminary evidence suggesting potential benefits for weight‐related health outcomes [116].

## 22 Physical activity and sedentary behavior

Promoting active lifestyles requires investment in infrastructure, workplace initiatives, and public awareness. Fiscal incentives, such as tax deductions for active commuting and higher parking fees, can encourage walking and cycling to work. Expanding pedestrian and cycling infrastructure, along with affordable public transport, supports daily movement and reduces car dependency. Schools should offer inclusive physical education, while workplaces can promote activity through walking meetings, fitness incentives, and active breaks. In health care, prescribing physical activity is a recommended strategy for preventing NCDs. Campaigns must ensure equitable access, including for marginalized groups, with particular attention to individuals with physical or mental health conditions who may need adapted environments and tailored support. Promoting EU‐wide and national physical activity guidelines is key to coordinated policy implementation, supported by regular public health campaigns highlighting the physical activity benefits (Table 3) [100, 117, 118, 119].

**Table 3:** European Code Against Cancer, 5th edition: recommendations for policymakers on physical activity and sedentary behavior.

Physical activity and sedentary behavior
• Implement fiscal incentives for all forms of active travel
• Promote and enable active public transportation for all ages and abilities, including vulnerable groups, by investing in suitable infrastructure
• Enhance urban planning policies to create safer, greener environments that encourage walking, cycling, and other mobility options in both urban and rural areas; strengthen the policy and design guidelines for public amenities, workplaces, and social housing to enable citizens with diverse abilities to have access and be physically active in and around buildings
• Introduce or reinforce physical education classes across the EU, with both curricula and infrastructure that allow for maximum inclusiveness
• Promote physical activity at work with initiatives and infrastructure standards
• Implement incentives for employers to provide opportunities for physical activity
• Introduce physical activity on prescription in primary care as a tool for prevention of noncommunicable diseases
• Work with vulnerable groups to address barriers to engaging in physical activity
• Update and promote EU‐wide and national physical activity guidelines
• Complementing the above‐mentioned policy measures, implement regular public health campaigns to raise awareness of the benefits of physical activity in the prevention of cancer

© 2026 International Agency for Research on Cancer / WHO. Used with permission.

References: • Council Recommendation of 26 November 2013 on promoting health‐enhancing physical activity across sectors. *OJEU*. 2013;**C354**:1–5. Available from: https://eur-lex.europa.eu/legal-content/GA/TXT/?uri=celex:32013H1204(01) [117]. • European Union Physical Activity Guidelines: Recommended policy actions in support of health‐enhancing physical activity. Brussels: European Commission; 2008. Available from: https://health.ec.europa.eu/document/download/97d6f94c-f4bd-4530-a488-c5f97f969a80_en?filename=2008_eu_physical_activity_guidelines_en.pdf [118]. • European Union Action Plan on Childhood Obesity 2014–2020. Brussels: European Commission; 2014. Available from: https://health.ec.europa.eu/publications/eu-action-plan-childhood-obesity-2014-2020 [100]. • Global action plan on physical activity 2018–2030: more active people for a healthier world. Geneva: World Health Organization; 2018. Available from: https://www.who.int/publications/i/item/9789241514187 [119].

### 22.1 Key policies

The Council Recommendation on Promoting Health‐Enhancing Physical Activity and the EU Physical Activity Guidelines emphasize active transport, access to recreational activities, and school‐ and workplace‐based physical activity [117, 118]. The EU Action Plan on Childhood Obesity promotes integrating movement into schools and ensuring equal physical activity opportunities for all children [100]. The Directive on Road Infrastructure Safety Management prioritizes pedestrian and cyclist safety, supporting active commuting [120]. The EU Save Energy Communication encourages walking and cycling as sustainable and health‐enhancing transport options [121]. The WHO Global Action Plan on Physical Activity calls for a whole‐of‐society approach, integrating physical activity into health care, workplaces, and urban planning [119], aligned with a salutogenic perspective that focuses on creating environments that actively promote and sustain health.

### 22.2 Creating enabling environments

Making physical activity an easy and appealing choice requires structural changes across sectors. Fiscal policies can encourage active commuting by increasing parking costs while offering tax deductions and mobility vouchers for walking, cycling, and public transport [117, 118]. Infrastructure improvements, such as expanded bike lanes, safer pedestrian crossings, and well‐connected transit networks, make active commuting more convenient and enjoyable [120, 122, 123]. Mandatory physical education ensures students have regular opportunities for movement [100, 118], while workplace policies can integrate active breaks, standing desks, and employer‐supported fitness programs. In health care, physicians prescribing physical activity as part of routine care can reinforce its role in disease prevention [119]. Public awareness campaigns should emphasize the risks of prolonged sedentary time and the benefits of regular movement. Tailored interventions must engage marginalized communities, addressing barriers and ensuring equitable access to sports, recreational facilities, and safe outdoor spaces.

### 22.3 Feasibility and resources

Several EU Member States offer compelling examples of successful physical activity promotion. The Netherlands, Denmark, and Germany have increased cycling rates through dedicated infrastructure investments, demonstrating the impact of urban planning on active transport [124]. Finland's tax incentives for workplace wellness encourage employers to support physical activity among staff [125]. Estonia's inclusive physical education policies ensure that students, regardless of ability, have equal access to structured movement programs [126]. Sweden's Physical Activity on Prescription program integrates exercise into health care, emphasizing its role in preventing chronic disease [127]. Europe's Sustainable Urban Mobility Plans highlight how integrating walking and cycling into urban planning can promote active lifestyles while addressing environmental, public health, and social challenges [128]. Collectively, these initiatives highlight how well‐planned interventions can foster a more active society.

## 23 Breastfeeding

Creating an enabling environment for breastfeeding requires comprehensive policies that protect, promote, and support mothers. Enforcing the WHO International Code of Marketing of Breast‐Milk Substitutes [129] is essential to prevent misleading promotions and ensure substitutes are used only when medically necessary. Paid maternity leave and flexible workplace arrangements enable exclusive breastfeeding for 6 months and continued breastfeeding thereafter. Employers should be encouraged to provide breastfeeding‐friendly workplaces, including lactation rooms and breastfeeding breaks. Public spaces must support breastfeeding without stigma. Training health‐care professionals, integrating breastfeeding education into prenatal/postnatal care, and establishing support networks can improve breastfeeding rates. Vulnerable groups, including single mothers, immigrants, adolescents, and those with prior challenges, require targeted support to ensure equitable access. In addition, regular public health campaigns are essential to raise awareness of breastfeeding and its health benefits for both infants and mothers (Table 4) [129, 130, 131, 132].

**Table 4:** European Code Against Cancer, 5th edition: recommendations for policymakers on breastfeeding.

Breastfeeding
• Ensure compliance with the International Code of Marketing of Breast‐Milk Substitutes, adopting and enforcing regulation to protect breastfeeding from inappropriate marketing of food products that compete with breastfeeding. Breast‐milk substitutes should be available when needed but should not be promoted
• Establish and enforce policies that ensure a sufficient duration of parental leave, as well as flexible working arrangements to enable working mothers to exclusively breastfeed their infants for 6 months and to continue thereafter
• Enact policies and introduce incentives for employers to provide breastfeeding‐friendly environments
• Encourage breastfeeding‐friendly policies and facilities in public areas, and protect the right of women to breastfeed whenever and wherever they need to
• Establish breastfeeding support networks. Train health‐care professionals to support new mothers in breastfeeding and make breastfeeding consultations accessible for all mothers
• Complementing the above‐mentioned policy measures, implement regular public health campaigns to raise awareness of breastfeeding and its health benefits for both the baby and the mother

© 2026 International Agency for Research on Cancer / WHO. Used with permission.

References: • International Code of Marketing of Breast‐Milk Substitutes. Geneva: World Health Organization; 1981. Available from: https://www.who.int/publications/i/item/9241541601 [129]. • Implementation guidance: protecting, promoting, and supporting breastfeeding in facilities providing maternity and newborn services: the revised Baby‐friendly Hospital Initiative 2018. Geneva: World Health Organization; 2018. Available from: https://www.who.int/publications/i/item/9789241513807 [130]. • Guidance on regulatory measures aimed at restricting digital marketing of breast‐milk substitutes. Geneva: World Health Organization; 2023. Available from: https://www.who.int/publications/i/item/9789240084490 [131]. • Protection, promotion and support of breastfeeding in Europe: a blueprint for action. European Commission, Directorate Public Health and Risk Assessment, Luxembourg, 2004. Available from: http://europa.eu.int/comm/health/ph_projects/2002/promotion/promotion_2002_18_en.htm [132].

### 23.1 Key policies

Several international and EU frameworks provide guidance on breastfeeding promotion. The WHO International Code of Marketing of Breast‐Milk Substitutes establishes restrictions on formula marketing to prevent commercial interests from undermining breastfeeding [129]. The WHO Baby‐Friendly Hospital Initiative (BFHI) outlines best practices for maternity and newborn care facilities, ensuring breastfeeding support is embedded in maternal health care [133]. Evaluations have shown that BFHI implementation is associated with higher breastfeeding initiation and duration rates [130]. To address evolving marketing tactics, the WHO Guidance on Digital Marketing of Breast‐Milk Substitutes targets online advertising strategies that discourage breastfeeding [131]. At the European level, the Protection, Promotion, and Support of Breastfeeding in Europe Blueprint for Action provides a comprehensive roadmap for strengthening breastfeeding policies, improving health‐care training, and raising public awareness [132].

### 23.2 Creating enabling environments

A supportive breastfeeding environment requires action across multiple sectors. Regulations on the marketing of breast milk substitutes must be strictly enforced to prevent misleading claims [129]. Workplace policies that guarantee sufficient maternity leave and breastfeeding accommodations empower mothers to continue breastfeeding after returning to work [130, 132]. Public facilities should include dedicated breastfeeding spaces, normalizing breastfeeding in everyday life. In health‐care settings, training for providers ensures that mothers receive the guidance they need [130], while prenatal and postnatal education fosters family wide support for breastfeeding. Additionally, community support networks play a vital role in helping mothers navigate challenges, with specialized outreach for marginalized populations to ensure that no group is left behind [132].

### 23.3 Feasibility and resources

Several EU countries have successfully implemented policies to enhance breastfeeding support. Finland's Family‐Friendly Workplaces Program incentivizes businesses to create breastfeeding‐friendly environments, supporting working mothers [134]. Spain's Breastfeeding‐Friendly Public Spaces Initiative designates safe areas for nursing mothers, helping to normalize breastfeeding in public [135]. Sweden has fully integrated the WHO Baby‐Friendly Hospital Initiative, ensuring maternity care facilities provide comprehensive breastfeeding support [136]. Germany's Breastfeeding Support Networks offer accessible consultations and peer‐led guidance, ensuring mothers have continuous assistance [137]. Together, these initiatives show how coordinated policies can remove barriers and support breastfeeding across diverse settings.

## 24 Conclusions

Preventing cancer is a key public health priority in the EU. ECAC5 recommends a healthy diet, weight management, physical activity, reduced sedentary behavior, and breastfeeding. Diets rich in whole grains, vegetables, legumes, and fruits, while limiting red and avoiding processed meat, can lower cancer risk. Reducing calorie, sugar, fat, and salt intake, especially from industrially processed foods and beverages of low nutritional quality such as many UPFs and SSBs, supports weight control, improves overall diet quality, and helps reduce cancers related to excess body weight and poor nutrition. Regular physical activity, less sedentary time, and breastfeeding benefit health and lower cancer risk. Individual action must be enabled by structural changes: affordable healthy food, supportive workplaces, and active urban infrastructure. Effective policies include taxation on unhealthy products, marketing restrictions, front‐of‐pack labelling, and investment in active transport. Addressing health inequalities is vital, as lower SES groups face greater cancer risks. Cross‐sector coordination can enable healthier choices and improve long‐term public health.

## Conflict of interest

All authors have no interests to declare. Where authors are identified as personnel of the International Agency for Research on Cancer/World Health Organization or the Organization for Economic Co‐operation and Development, the authors alone are responsible for the views expressed in this article and they do not necessarily represent the decisions, policy, or views of the International Agency for Research on Cancer/World Health Organization, or the Organization for Economic Co‐operation and Development or its member countries. Likewise, where authors are affiliated with the European Commission, the contents of this publication do not necessarily reflect the position or opinion of the European Commission.

## Author contributions

MFL and IB were responsible for writing the first version of the manuscript. All authors gave critical revisions on the intellectual content of the manuscript and approved the final manuscript.

## Supporting information


**Annex S1.** European Code Against Cancer, 5th edition. © 2026 International Agency for Research on Cancer / WHO. Used with permission.

## Data Availability

The data that supports the findings of this study are available in Tables 1, 2, 3, 4 and Annex S1 of this article.
